# The Transfer of Object Learning after Training with Multiple Exemplars

**DOI:** 10.3389/fpsyg.2016.01386

**Published:** 2016-09-21

**Authors:** Annelies Baeck, Karen Maes, Chayenne Van Meel, Hans P. Op de Beeck

**Affiliations:** Brain and Cognition, University of LeuvenLeuven, Belgium

**Keywords:** perceptual learning, object learning, transfer, exemplar, training

## Abstract

Object recognition improves with training. This training effect only partially generalizes to untrained images of the trained objects (new exemplars, orientation,…). The aim of this study is to investigate whether and to what extent the learning transfer improves when participants are trained with more exemplars of an object. Participants were trained to recognize two sets of stimuli using a backward masking paradigm. During training with the first set, only one exemplar of each object was presented. The second set was trained using four exemplars of each object. After 3 days of training, participants were tested on all the trained exemplars and a completely new exemplar of the same objects. In addition, recognition performance was compared to a set of completely new objects. For the objects of which four exemplars were used during training, participants showed more generalization toward new exemplars compared to when they were only trained with one exemplar. Part of the generalization effect extended to completely new objects. In conclusion, more variation during training leads to more generalization toward new visual stimuli.

## Introduction

The topic of generalization is key in the literature of perceptual learning. It is often handled in a relatively simplistic way: most studies do not manipulate the variation within the trained set, and look for generalization in a binary way (is it there or not) or at most use a unidimensional approach (degree of generalization as one dimension, for example the specificity index from Ahissar and Hochstein, 1997). For instance, Furmanski and Engel (2000) trained participants to recognize a set of pictures of common objects. After a training period, they found that the learning effect was specific to the trained objects (no generalization), but did transfer to different sizes of the trained object pictures (generalization does happen). Specificity or generalization of the training effect has been investigated for a wide range of properties, such as orientation (e.g., Fiorentini and Berardi, 1981; Baeck et al., 2012) visual field location (e.g., Karni and Sagi, 1991), direction of movement (e.g., Ball and Sekuler, 1982, 1987), spatial frequency (e.g., Yu et al., 2004), exemplar (Baeck et al., 2012), and contrast (Yu et al., 2004).

More recently, researchers started to investigate properties of the training task and their influence on the learning transfer. For example, Jeter et al. (2010) found generalization for orientation and location at the beginning of the training process, whereas more specificity for these trained properties was found after an extensive training period. Other relevant manipulations include the training procedure, such as the double training procedure (Wang et al., 2012) or training-plus-exposure (Zhang et al., 2010), task difficulty (Garcia et al., 2013), task precision (Jeter et al., 2009) and whether or not stimulus-specific or stimulus-general rules are promoted during training (Green et al., 2015).

In the current study, we aim to investigate the effect of the variation of the training set on the learning transfer. In a previous experiment, we found that object learning generalizes only partially over changes in exemplars (Baeck et al., 2012). In that experiment, we trained participants with one exemplar of a set of objects, and then tested them on the trained and new exemplars of that set of objects. When multiple exemplars are presented during training, participants might learn a more general or varied template of the objects, thereby possibly enhancing the learning transfer to new exemplars. To test this idea, in the current experiment we vary the number of training exemplars per object and investigate the influence of the variation of the training set on the generalization of the training effect toward new exemplars of the trained objects and completely new objects.

There is already some evidence for the hypothesis that the generalization of learned tasks with objects improves when participants are trained with more exemplars of an object. When rats are trained to discriminate between complex objects despite substantial variation in the appearance of the training objects (i.e., changes in size, view, and lighting), the learning effect did transfer to new variations of the trained objects (Zoccolan et al., 2009). However, when the animals are trained with stimuli that do not vary in their appearance, there is no generalization to new stimulus variations (Minini and Jeffery, 2006). In a face recognition task, participants showed better performance toward new exemplars of the learned faces after a short training with multiple exemplars than when exemplars per face were constrained (Murphy et al., 2015). In all these studies, one could argue that the learning as well as the generalization involves learning associations between objects and decision rules. From that perspective the question of generalization in these studies relates to the extensive literature on category learning (Komatsu, 1992; Ashby and Maddox, 2005). Also in that literature there is evidence that the generalization of learned category rules to new exemplars is influenced by the variation of category exemplars which were experienced during training (Homa and Vosburgh, 1976; Minda and Smith, 2001).

In the present study, we extend this research to a visual learning paradigm in which the challenge to subjects is not so much to learn to associate objects with responses, but rather to improve the perception of the objects. We test whether exposure to more exemplars during training will improve the perception of the objects. In this experiment, the term ‘exemplar’ refers to the specific picture of a particular object (for example a picture of one specific umbrella). All different exemplars belong to a particular type of object (in this example, the object ‘umbrella’). We investigated whether in a typical perceptual learning experiment, in which we train participants over multiple days to recognize images of common objects, more transfer of the learning performance toward new images of these objects will occur when participants are trained with more exemplars. Various aspects of our results show that the variation of exemplars seen during training does influence the degree of generalization during testing.

## Materials and Methods

### Participants

Initially 30 naive subjects participated in the experiment. After initial results showed smaller effect sizes (improvement of learning over days) than expected based on previous research (Baeck and Op de Beeck, 2010; Baeck et al., 2012, 2014; Van Meel et al., 2016), an additional group of 15 participants were tested. One participant did not participate in all sessions of the experiment and these data were not analyzed. All remaining 44 participants (six male, average age 22.9, age range 18–54) had normal or corrected to normal sight. Before every session, they signed an informed consent. The experiment was approved by the faculty of Psychology and Educational Sciences (KU Leuven).

### Apparatus

Stimuli were presented using Psychtoolbox [(Brainard, 1997) in MatLab 6.0 (Mathworks, Inc.)] on a Dell desktop computer. The room was darkened during stimulus presentation. Viewing distance was approximately 75 cm.

### Stimuli

**FIGURE 1:**
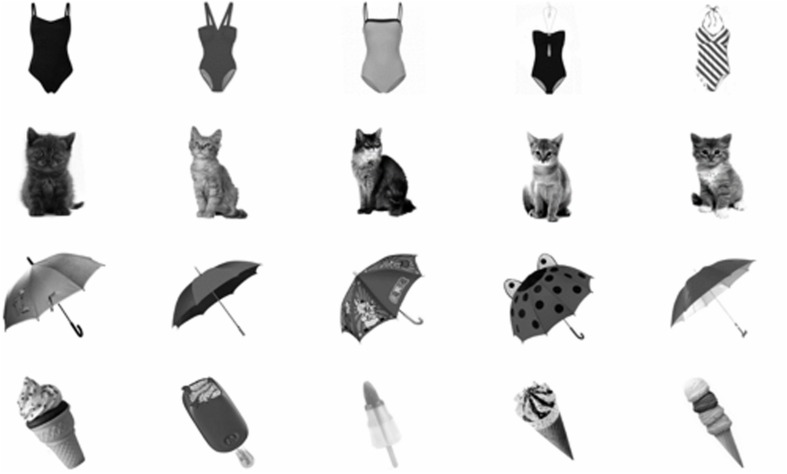
**Example stimuli: Five exemplars per common object**.

The stimulus set comprised 300 gray scale images of common objects on a white background. Part of the stimulus set was previously used in Baeck et al. (2012), but most images were created for the purpose of this experiment. Sixty common objects (e.g., umbrella, bathing suit,…) were selected based on easy recognition and few available synonyms. For each object, five distinct exemplars were created (**Figure 1**). The exemplars were matched for orientation. The stimuli were divided in three sets of 20 objects with five exemplars per object (**Supplementary Figure S1**). The sets were balanced with respect to the proportion of living/non-living objects and difficulty level (estimated through pilot testing with one of the authors). Image size was 567 × 567 pixels (12.5 visual degrees). Stimulus contrast was reduced to 12.5% of the original contrast to increase recognition difficulty level. Masking stimuli were created with small fragments (70 × 70 pixels) of different object pictures. This type of mask is effective in masking the stimulus in a backward masking paradigm (Op de Beeck et al., 2007). The masking stimuli were the same size as the object stimuli. All stimuli were gamma corrected in order to create a linear luminescence range. Given that this correction reduced the overall contrast of the images, an inverse gamma-correction was applied to the masking stimuli in order to create a more robust masking effect.

### Learning Paradigm

Each trial started with a fixation dot, followed by an object image and three consecutive masks (each 250 ms) to prevent further visual processing. The stimuli and masks were presented at slightly different random locations close to the center of the screen (a maximum deviation of 1.1 visual degrees from central presentation). Participants were then instructed to identify the object by typing the first three letters of the object name. The duration of the object image was adaptive. During the first trial, the object was presented for 120 ms. The duration on the following trials depended on the performance of the participant and was determined based on a interleaved two-down-one-up staircase procedure. After two correct consecutive answers, the presentation duration of the object decreased by 10 ms (one frame rate at 100 Hz), increasing the difficulty level. When participants did not correctly identify the object, the presentation duration of the object image in the next trial was increased by 10 ms. Two staircases of 40 object images each were interleaved in every training run. Participants received feedback after every trial. In case of a wrong answer, the correct object name was provided.

### Procedure

**FIGURE 2:**
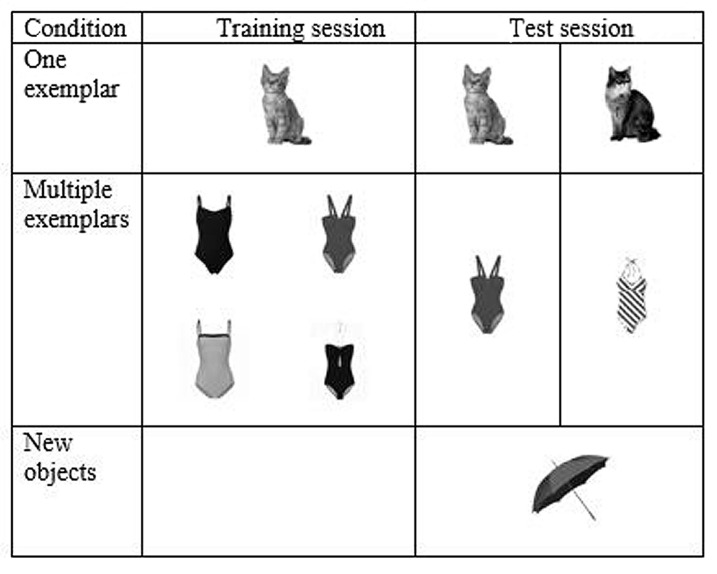
**Procedure during training and test days with example stimuli.** Every stimulus set is represented by one object. During training, the participant is trained with one exemplar per object of stimulus set 1 (here: the cat) and four different exemplars of stimulus set 2 (here: the four different bathing suits). During testing, recognition performance is tested for the trained exemplars (trained cat and one trained bathing suit image), a new exemplar of the trained stimulus sets (untrained cat and untrained bathing suit image) and an exemplar of stimulus set 3 (here: the umbrella).

Participants were trained on three consecutive days. After the training period, a test day followed. All training and testing was finished within 1 week. The training and testing procedure is visualized in **Figure 2**. Every participant was trained with two sets of objects. For one set of objects, only one of the exemplars was selected and used during training. This is called the one exemplar condition. In every training run of this condition, participants were presented four times with each exemplar. For the second set of objects, the participants were trained with four of the five exemplars of each object. This is further called the multiple-exemplars-condition. Within one training run all 80 exemplars (four exemplars for each of the 20 objects within one stimulus set) were presented once.

Each of the three training sessions started with a preview of the two stimulus sets presented in the training runs. During the preview one exemplar of every object was presented for 2 s with the corresponding object name. This preview procedure has also been applied in previous perceptual learning studies using a similar paradigm (Furmanski and Engel, 2000; Baeck et al., 2012, 2014). For the objects in the one exemplar condition, the exemplar during the preview was the same exemplar as the one presented in the training runs. For the object in the multiple exemplars condition, one of the exemplars presented during training was selected. This selected exemplar remained the same in every preview during the training procedure. Participants completed eight runs per training session, four runs per condition. One exemplar condition and multiple exemplars condition runs were alternated and counterbalanced over participants.

During the test session on the fourth day, recognition performance was tested for five different conditions: (1) trained exemplars from the set of the one exemplar condition, (2) untrained exemplars from the stimulus set used in the one exemplar condition, (3) trained exemplars from the stimulus set of the multiple exemplars condition, (4) untrained exemplars from the stimulus set of the multiple exemplars condition, and (5) exemplars from a completely new set of objects. To minimize differences between the conditions in the test session, we only tested one trained exemplar per object presented in the multiple exemplars condition. This exemplar was the same as the exemplar selected for the preview during training. For all five conditions presented in the test run, a preview was presented at the beginning of the test run.

The exemplar presented during preview, the exemplars selected for training and testing, the allocation of the different stimulus sets to the different conditions and the order of the conditions within one session were counterbalanced between participants. Daily training sessions lasted approximately 45 min, the last test session approximately 1 h.

### Data Analysis

Individual perceptual thresholds, i.e., the presentation time at which participants could still accurately recognize the objects, were calculated using the endpoints of the staircases. A lower threshold indicates better task performance. For every participant, the endpoints were averaged per condition and per day.

The addition of extra participants after observing an unexpectedly small training-related improvement in the first 30 participants (see sections Participants, Training effect, and Discussion) might influence the interpretation of the significance of the test results (see e.g., Armitage et al., 1969). We ran simulations to investigate the effect of our decision to test an additional group of 15 participants after non-significant effects (0.05 < *p* < 0.20) were found with the original group of 30 participants. After 10000 simulations, *p*-values smaller than 0.05 were found in 6.98% of the cases. To reduce this to 5% of the cases, a more conservative critical threshold of α = 0.036 to which the significance levels (*p*-values) of our tests are compared will be used.

## Results

### Training Effect

A repeated measures ANOVA was used to test for the effect of session (days 1–4) and condition (trained exemplars of the one exemplar and multiple exemplars conditions). Overall, performance did improve over time [*F*(1,43) = 88.100, *p* < 0.001]. To investigate whether the training effect diminishes over time, difference scores between the different days were calculated. A main effect was found [*F*(2,86) = 9.914, *p* < 0.001]. Further testing showed that the performance gain between the first and second day was indeed larger than the performance gain between days 2 and 3 [*t*(43) = 3.787, *p* < 0.001], while no difference in improvement was found between the difference scores for days 2 and 3 versus days 3 and 4 [*t*(43) = 0.626, *p* = 0.535]. Although the training-related improvements were strongly significant, it is useful to note that they are very small in magnitude, with average improvements around 8 ms. This is smaller than one step in the adaptive procedure, and smaller than the 3-day improvements in similar perceptual learning experiments (Baeck and Op de Beeck, 2010, 18 ms; Baeck et al., 2012, 11 ms).

**FIGURE 3:**
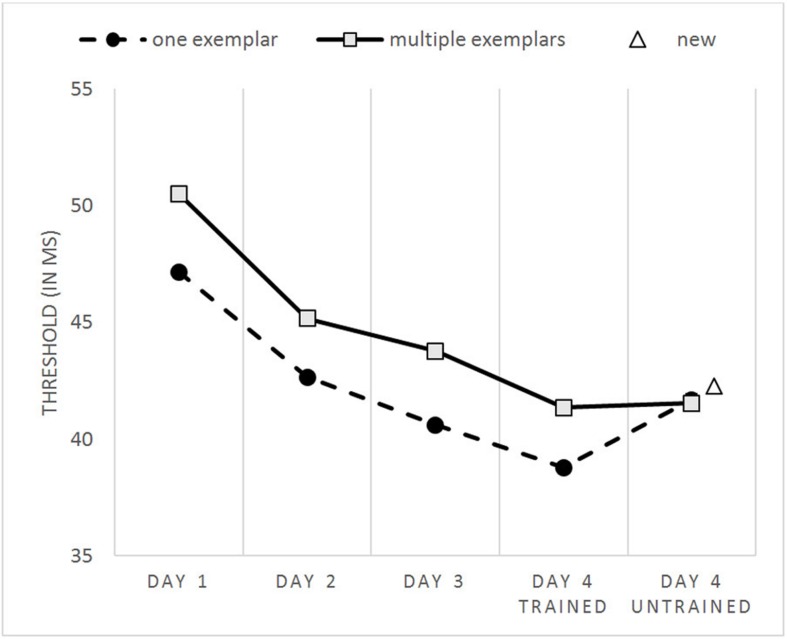
**Performance thresholds plotted as a function of the session and condition**.

**Figure 3** shows lower thresholds, and thus a better performance for the one exemplar condition compared to the multiple exemplars condition during training. This is not surprising, as every single exemplar in the one exemplar condition was presented four times more frequently than the exemplars in the multiple exemplars condition. This main effect for condition was significant [*F*(1,43) = 39.254, *p* < 0.001]. No interaction between time and number of training exemplars was found [*F*(3,129) = 0.418, *p* = 0.740].

### Transfer to New Exemplars

Within the one exemplar condition, recognition performance for the trained exemplar is better than for the untrained exemplar [*t*(43) = 3.963, *p* < 0.001] on the test day. Participants thus learn to recognize the particular object image they are trained with. When comparing performance to the untrained exemplar with performance level on the first day, a significant difference was found [*t*(43) = 6.474, *p* < 0.001], indicating that part of the training effect did transfer to the new exemplar.

In the multiple exemplars condition, the threshold for the trained exemplar is not significantly different from the threshold for the untrained exemplar [*t*(43) = 0.201, *p* = 0.841]. This differs from the one exemplar condition, and is a first indication that more transfer to new exemplars can be found when participants are trained with more exemplars.

The critical test to investigate whether there is more transfer of performance to new exemplars when participants are trained with four exemplars versus one exemplar was performed with a 2 (trained versus untrained) × 2 (trained with one versus four exemplars) repeated measures ANOVA. As expected, a main effect of familiarity was found [*F*(1,43) = 8.803, *p* = 0.005]: performance for trained exemplars is better than performance for untrained exemplars. The main effect of condition was in the expected direction (better performance in the one exemplar condition) but did not reach significance [*F*(1,43) = 3.490, *p* = 0.069]. Most importantly, we did find a significant interaction between familiarity and training condition [*F*(1,43) = 5.192, *p* = 0.028], confirming that more transfer of performance to new exemplars can be found when participants are trained with multiple exemplars.

### Transfer to New Objects

We further investigated whether, in addition to a transfer of performance to new exemplars of trained objects, a generalization of the learning effect could be found for completely new objects. When comparing the performance for completely new objects with the thresholds on the first day, a significant difference was found independently of which condition was used as the benchmark for the first day [one exemplar condition: *t*(43) = 4.923, *p* < 0.001; multiple exemplars condition: *t*(43) = 8.697, *p* < 0.001]. Thus at least part of the training effect did transfer to the new objects. For both conditions, performance for the new exemplar of the trained objects did not differ from the performance for the exemplars of the completely new objects [one exemplar condition: *t*(43) = 0.661, *p* = 0.512; multiple exemplars condition: *t*(43) = 0.939, *p* = 0.353]. Participants thus showed a similar amount of generalization of the learning effect toward new exemplars of trained objects and new exemplars of new objects. When comparing to performance of trained exemplars, perceptual thresholds for the new objects were higher (and thus showed a worse performance) compared to the trained exemplar of the one exemplar condition [*t*(43) = 3.601, *p* = 0.001]. However, no significant difference was found between thresholds for the new objects and trained exemplars in the multiple exemplars condition, thus showing a complete transfer of the learning effect toward new objects.

This result pattern is surprising, as in a very similar learning experiment, a better performance for new exemplars of trained objects was found compared to completely new objects (Baeck et al., 2012). Furthermore, our previous studies investigating perceptual learning with complex objects using the same learning paradigm found partial (Baeck et al., 2014) or even complete specificity for the trained objects (Baeck and Op de Beeck, 2010; Baeck et al., 2012; Van Meel et al., 2016). The exact parameters differed between different studies, but all trained participants with a set of 20 objects during multiple days, and tested afterward the threshold for the trained set and a completely new set of objects. This is most comparable to the one exemplar condition in the current experiment, where indeed partial specificity is found, but also in this condition the amount of generalization seems larger compared to the previous studies. In general, more generalization toward new stimuli seemed to occur in the current experiment compared to our previous perceptual learning experiments. We therefore decided to directly compare the object specificity between this study and the previous learning studies.

**FIGURE 4:**
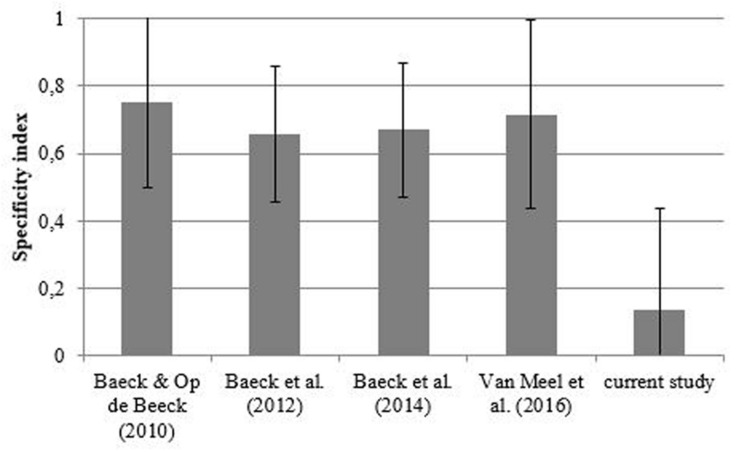
**Mean specificity indices in the different perceptual learning experiments with complex objects.** Errors bars represent the standard error of the mean (SEM).

To compare thresholds between the different studies, we first calculated individual specificity indices (as also used in Baeck et al., 2012) by dividing the difference in threshold value on the last day for the new objects and the trained exemplars by the total learning effect: (new objects-trained exemplars)/(day 1 performance – trained exemplars). A higher index value indicates a higher degree of object specificity. From the study of Van Meel et al. (2016) only the sham group was included. From the current experiment, we only included the one exemplar condition, as this condition is most similar to the other learning studies, where only training with 20 objects occurred. Note that including the four exemplar condition would make the specificity index even lower (so it is a conservative choice not to include the four exemplar condition). Both in the study of Baeck et al. (2012) and the current experiment data from one participant were discarded because the specificity index could not be calculated (division by zero). For the current study, an average specificity index of 0.13 was found, while the specificity index values for the previous learning studies fall between 0.66 and 0.75 (**Figure 4**). The difference between the object specificity in the current experiment and previous experiments was nearly significant [*t*(108) = 1.946, *p* = 0.054].

## Discussion

In this experiment, we trained participants over multiple days to learn to recognize two sets of images of common natural and manmade objects. For one set, participants were only trained with one exemplar per object. More variation in training was provided for the second object set, namely four exemplars per object. As expected, results showed that more variation in the training set does lead to more transfer of the learning effect toward new exemplars of the trained objects. More surprisingly, we also found a considerable amount of transfer to completely new objects.

In the one exemplar condition, we found a partial generalization toward the new exemplar of trained objects. This is in accordance with earlier behavioral findings (Baeck et al., 2012). Even though neurons are sensitive toward differences between exemplars of an object (Op de Beeck et al., 2001; Panis et al., 2008), participants can generalize their behavioral response toward new exemplars in a variety of tasks (for a review, see Ashby and Maddox, 2005). Through the object learning process, participants either learn rules defining the critical features of the object, possibly through a neural system involving the prefrontal cortex, or learn to recognize particular exemplars to which new exemplars can be compared, possibly reflecting learning within the visual system (Rouder and Ratcliff, 2006). As a result, the learning effect can be generalized to previously unseen exemplars of the same objects. More variation during the training procedure can enhance this process, either by better defining the rules to perform the recognition task (task-related learning of the prefrontal system) or by providing more exemplars to compare the new exemplar with (learning of the visual system). With the current experiment, we cannot distinguish between these options. When we compare the amount of transfer toward new exemplars in the one exemplar and multiple exemplars condition, we indeed find more generalization toward new exemplars of trained objects when participants were presented with a larger variety of exemplars during the training procedure, even to the point that the recognition performance is as good for new exemplars as for the trained exemplars. This enhanced generalization is in accordance with earlier findings in studies which tested how subjects generalize object-response associations to new object exemplars (Zoccolan et al., 2009; Murphy et al., 2015).

A more detailed comparison of the one exemplar and four exemplar training suggests that the improved generalization to novel exemplars might be the consequence of two processes. A first process is a direct improvement of generalization, possibly because training with more exemplars optimizes the inter-/extra-polation from old to new exemplars (Poggio and Edelman, 1990). A second process is a decrease of performance during training for the trained exemplars in the four exemplar condition (main effect of condition during training), as if a larger variety of stimuli during training makes it more difficult for subjects to perform the task. This decrease in performance on the trained stimuli will diminish the difference between trained and untrained stimuli, and as such indirectly improve generalization as assessed by computing the difference in performance between trained and untrained. Both these two processes seem to be implicated in our finding that generalization improves when training involves more stimulus variety.

In addition, a considerable transfer of performance toward images of completely new objects was found, more than previously found in comparable perceptual learning studies. It is important to note that the nearly significant difference in learning transfer between the perceptual learning studies cannot be explained by the amount of training. In general, more training leads to better results, but the learning follows a negatively accelerated curve (Ritter and Schooler, 2001). This effect is again evidenced by the results in our study. With regard to the relation between amount of training and transfer toward new stimuli, evidence is found that more learning transfer occurs at the beginning of the training process, while extensive training with specific stimulus properties increase the specificity of the learning process (Jeter et al., 2010). The current study trained participants with 1920 trials spread over three training sessions during three different days within 1 week. This is well within the range of training amount in the other studies used in the comparison in Result section 3.3:3200 trials over five training days (Baeck and Op de Beeck, 2010; Baeck et al., 2012), 2560 trials in 4 days (Baeck et al., 2014), and 1080 trials in three training days (Van Meel et al., 2016). The training is also sufficiently long, and participants show a clear training effect (**Figure 3**). Therefore, it cannot be explained by the finding of Jeter et al. (2010).

Even though the studies differ in a range of aspects related to the research question at hand and other small changes with regards to the stimulus set, training duration and precision of the measurement (frame rate), in all of these studies the same object recognition task was used with a backward masking paradigm and training occurred over multiple days. The critical difference between the current and the previous perceptual learning studies seems to be the number of exemplars presented during training. While in the previous experiments participants were only trained with 20 different images, participants were presented with 100 different object images/exemplars in the current study (one set of 20 objects with one exemplar per object, plus one set of 20 objects with four exemplars per object). The comparison does give a good first indication that more variation in the training set does lead to more transfer of performance toward completely new objects, but a more consistent comparison between the two conditions would be useful in the future.

The marked generalization even to new objects diminishes the range of values which can be expected for novel exemplars of trained objects. If even a completely new object is associated with performance close to the performance for a trained exemplar of a trained object, then we cannot expect a large difference between trained and novel exemplars of trained objects. This is also a reason why the effect size of differences between trained and novel exemplars, and how this interacts with condition (one or four exemplars during training), is low in the current study. One could say that the very effect that we were after in the present study, better generalization when trained with more exemplars, has turned against us by decreasing our ability to differentiate performance between trained stimuli and any type of new untrained stimuli.

In the present study, we seem to have taken this perceptual learning paradigm to its limit. Once participants are trained with a relatively large number of objects, very little object specificity remains. As a consequence, the findings more and more reflect a general improvement in the task independent of whether an object image was shown during training or not. The stimulus specificity which is often seen as a hallmark in perceptual learning is lost when a large variety of stimuli is used during training. The consequence is an overall improvement in task performance due to training, with only minor remaining differences between trained and untrained stimuli. Whether or not such generalization is beneficial, depends upon the goals of the training. In applications outside the laboratory generalization is often wanted (e.g., Deveau et al., 2014), thus our study adds to the available evidence that generalization improves by including a large variety of stimuli during training. Whether these findings extend to more naturalistic settings and whether this effect is modality-specific remains to be studied.

## Author Contributions

AB, KM, and HO contributed to the conception and design of the study. Data collection was performed by KM and CM and data analysis by AB and KM. All authors contributed to drafting the work.

## Conflict of Interest Statement

The authors declare that the research was conducted in the absence of any commercial or financial relationships that could be construed as a potential conflict of interest.

## Supplementary Material

FIGURE S1 **Complete stimulus sets with the corresponding object names in Dutch and the English translation**.
