# Supplementary figures and images for: The Transfer of Object Learning after Training with Multiple Exemplars
Source: Front Psychol. 2016 Sep 21;7:1386. doi: 10.3389/fpsyg.2016.01386 (PMC5030234; doi:10.3389/fpsyg.2016.01386)

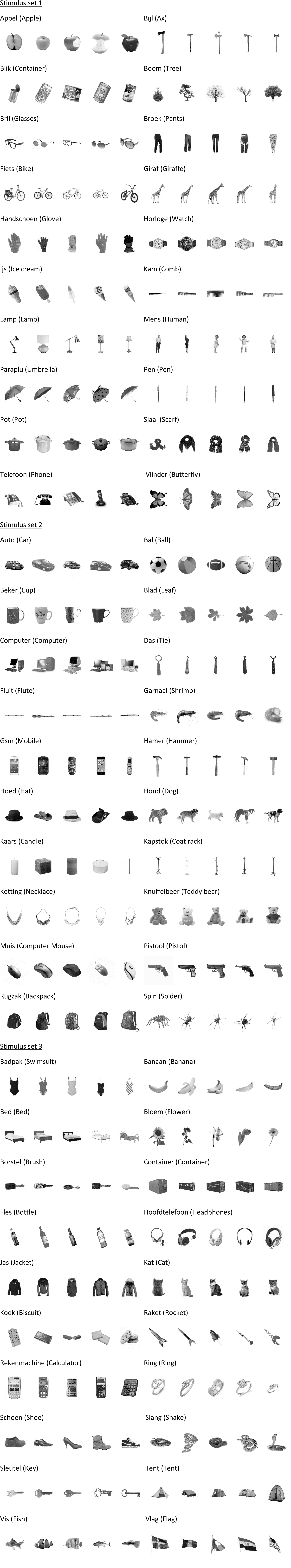

Supplement: FIGURE S1 — Complete stimulus sets with the corresponding object names in Dutch and the English translation. [file Image_1.JPG]
